# Measurable (Minimal) Residual Disease in Myelodysplastic Neoplasms (MDS): Current State and Perspectives

**DOI:** 10.3390/cancers16081503

**Published:** 2024-04-15

**Authors:** Linsheng Zhang, George Deeb, Kristin K. Deeb, Colin Vale, Deniz Peker Barclift, Nikolaos Papadantonakis

**Affiliations:** 1 Department of Pathology and Laboratory Medicine, Emory University School of Medicine, Atlanta, GA 30322, USA; 2 Department of Hematology and Medical Oncology, Winship Cancer Institute of Emory University, Atlanta, GA 30322, USA

**Keywords:** myelodysplastic neoplasm, measurable residual disease, mutation profile, cytogenetics, error correction sequencing, single cell sequencing, flow cytometry, hematopoietic stem cell transplantation

## Abstract

**Simple Summary:**

Myelodysplastic Neoplasms (MDS) are a type of blood cancer presenting as ineffective production of blood cells, even though the bone marrow appears active. Traditionally, the response to treatment has been studied by examining blood cell counts, morphologic features, and chromosomal changes. Recently, the ability to evaluate neoplastic status has been significantly improved by the development of highly sensitive flow cytometry and molecular assays, allowing for the identification of residual low-level neoplastic components. In this review, we discuss the evolving concept of measurable (minimal) residual disease (MRD) in MDS in clinical practice, elaborate on the laboratory methods utilized to identify low-level neoplasms, and provide an overview of the published studies correlating MRD with the clinical outcomes of MDS patients.

**Abstract:**

Myelodysplastic Neoplasms (MDS) have been traditionally studied through the assessment of blood counts, cytogenetics, and morphology. In recent years, the introduction of molecular assays has improved our ability to diagnose MDS. The role of Measurable (minimal) Residual Disease (MRD) in MDS is evolving, and molecular and flow cytometry techniques have been used in several studies. In this review, we will highlight the evolving concept of MRD in MDS, outline the various techniques utilized, and provide an overview of the studies reporting MRD and the correlation with outcomes.

## 1. Introduction

Myelodysplastic neoplasms (MDS) are a heterogenous group of myeloid neoplasms characterized by cytopenias and morphological abnormalities in the bone marrow [1] that predominantly affect elderly patients [2]. While the overwhelming majority of patients have cytogenetic or mutational aberrations [3,4], patients may range from having mild cytopenias, requiring only periodic monitoring, to more profound cytopenias with increased blasts necessitating intervention, such as chemotherapy or allogeneic bone marrow transplants (alloHSCT) [5,6,7,8].

Response to treatment has been assessed by peripheral blood count assessments, bone marrow morphological reviews, and cytogenetic evaluations [9,10,11,12]. In recent years, the advancement of high-throughput genomic technologies and high sensitivity multiparameter flow cytometry (MFC) allows for monitoring of MDS beyond morphological and karyotypic assessments [12].

Criteria for MDS response did not formally included measurable (minimal) residual disease (MRD) in the 2006 modification of the International Working Group (IWG) response criteria [11]; however, the IWG 2023 response criteria for high-risk MDS included an MRD-negative response as a provisional response category [12]. The panel recognized that detecting MRD in high-risk MDS had not been standardized and made universally available.

The aims of this review are to discuss different MRD techniques and their strengths and limitations, as well as to provide an overview of MRD testing in MDS studies as well as the emerging technologies that may impact the monitoring of MDS in the future.

## 2. Molecular, Genomic-Based Detection of MRD in MDS

Detection of genetic abnormalities and mutation profiling have been the cornerstones in the diagnosis, risk stratification, and guiding treatment decisions for MDS, especially after the discovery that some recurrent mutations are relatively characteristic of MDS [13,14].

**Figure 1:**
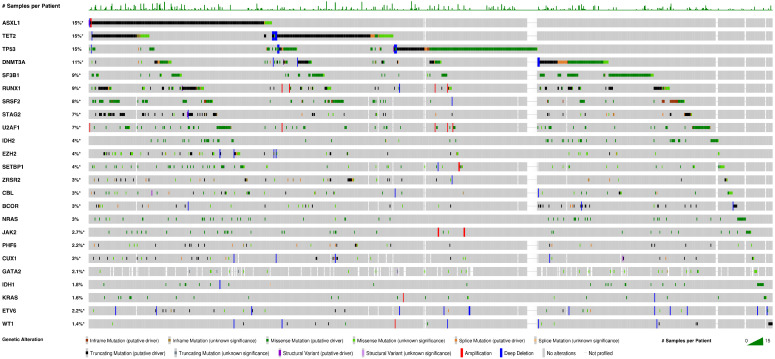
Top mutations in myelodysplastic neoplasm (MDS).

By array-based genetic testing and next generation sequencing (NGS), up to 90% of MDS patients harbored one genetic abnormality or mutation [15], and 74% carried at least one mutation detectable by an NGS target gene panel, the vast majority of which were found in a limited number (<50) of genes [16]. The mutation landscape of MDS is well represented in the AACR Project GENIE Cohort v15.0-public dataset (see Figure 1) [17].

A query of the AACR Project GENIE Cohort v15.0-public [17] identified 2816 samples from 1724 MDS patients. Twenty-four genes with mutations detected in >1% of patients are displayed as mutations per patient. In this dataset, 54% (925 of 1724) of patients had at least one mutation in these 24 genes. The color codes for different types of mutations are indicated at the bottom of the OncoPrint image. Only cases with mutations are displayed. Image created by cBioPortal for GENIE [18,19,20].

Data link: https://genie.cbioportal.org/study?id=65be692383e9543d618f0df0 (accessed on 3 February 2024). # Samples per Patient: The number of samples tested per patient is represented by the height of the green bar. * not all samples are profiled. 

The NGS methods currently available in clinical laboratories include sequencing by synthesis derived from the chemistry, which is similar to traditional Sanger or pyrosequencing methods. Numerous clones of molecules (clusters or beads) from a single short DNA fragment with added adaptors are sequenced to generate signals for sequencing reads. Because adaptors are added to the original DNA fragments, multiple patients/samples and different molecules can be labeled separately by different adaptor sequences, allowing sample multiplexing and molecular barcoding of each DNA fragment using a unique molecular index (UMI). Due to the limited sequencing read length (cycles of sequencing by synthesis), NGS methods are unable to detect large structural variants (SVs) and copy number variants (CNVs). DNA-based NGS is also not optimal for detecting fusion genes. To create molecular clones for sufficient signals to generate sequencing reads, short DNA fragments are amplified by PCR during library preparation or cluster generation. In addition, sequencing by synthesis requires a polymerase reaction to add nucleotides to extend the strand based on the template. These processes generate errors intrinsic to the DNA polymerase, which may also be related to the template sequence. The combined error rate of sequencing by synthesis-based NGS is approximately 1% for single nucleotide variants (SNVs) [21,22]. With appropriate informatics analysis, the specificity of larger indels may be better, as creating errors of larger indels is biochemically unlikely [23,24]. A significant disadvantage of NGS is that the detection of indels is generally size-limited [25]. Modification of the wet lab method and bioinformatics analysis is required to detect large indels and structural variants [21]; however, it is more challenging for MRD detection, where excellent analytical sensitivity is critical. The techniques and methodology for MRD testing are more established in AML [26,27].

To achieve analytical sensitivity to assess deep molecular responses, error correction by UMI and bioinformatics analysis during variant calling, as well as separate tagging of forward and reverse strands (duplex sequencing) are recommended for MRD detection [22,28]. UMIs are unique nucleotide sequences added to the fragments to be sequenced either by adaptor ligation or tagged at the 5′-side of the PCR primers to be added before or during the first round of amplification so that all sequencing reads generated from each original template molecule (a read family) can be recognized and processed to remove the errors generated by PCR or sequencing reactions. A high-read depth, large-read family size, and a minimal number of variant reads are required for sufficient sensitivity and specificity. The technical details regarding NGS-MRD recommended by the European LeukemiaNet MRD Working Party [29] for AML have been made available. For MRD testing in AML, the MRD threshold was provisionally defined at 0.1% variant allele frequency (VAF) [29]. Encouragingly, with appropriate technical optimization [30], the detection sensitivity of NGS can reach 1.3 × 10^−6^, which affords an even higher sensitivity. However, although, by extrapolation, AML MRD criteria may be also applicable to MDS, further studies will be required to determine the optimal cut off for molecular remission for MDS.

There are also important differences and considerations in a similar approach to AML MRD monitoring within the context of MDS.

(1) In AML, the leukemic blasts (myeloblasts, abnormal promyelocytes, monoblasts, and promonocytes) are the major type of neoplastic cells. In contrast, the MDS neoplastic cells comprise varied percentages of blasts (up to 19%) with a variable retained ability of differentiation and maturation. While clearance of blast-specific mutations or transcripts (such as *NPM1*, *RUNX1::RUNX1T1*, *CBFB::MYH11*) is indicative of a molecular response in AML, the molecular response in MDS is not standardized and difficult to define, especially for low-risk disease [12]. Such a molecular response may require clearance of mutations harbored in a heterogenous group of clones and subclones of the neoplastic cells, with the potential exception of *DNMT3A*, *TET2*, and *ASXL1* (DTA) mutations, which may be considered to represent age-related clonal hematopoiesis of indeterminate potential (CHIP) [31,32].

(2) A significant percentage of AML blasts carry specific driver mutations or fusion genes that PCR assays afford a high-level sensitivity, whereas there are no mutations that are diagnostic or specifically represent the neoplastic clonal process of MDS (with the exception of *SF3B1* or multi-hit *TP53* in the setting of not meeting morphological criteria for AML). A high percentage of patients with MDS carry more than one MDS-associated mutation, and there is significant clonal diversity [33,34,35,36] in the neoplastic population of MDS. A quantitative PCR or digital PCR-based assay can be considered when only a few mutations known at the time of initial diagnosis or only clinically critical mutations, such as *TP53* mutations, are tested for MRD monitoring [37]. However, a methodology that can detect and monitor aberrations in multiple genes is generally preferred for MDS. NGS-based MRD testing is an attractive platform that could be applied to most MDS cases.

The level of sensitivity of the assay used at diagnosis is a key determinant of using the patient-specific mutation profile to monitor MRD. If the assay cannot detect mutations at a low VAF, mutations below the level of detection may be missed at initial diagnosis. Furthermore, clonal evolution is also frequent in the disease process of MDS [34,38]. Therefore, during subsequent assessments, it could be difficult to determine if a newly detected mutation represents a pre-existing aberration, that is now above the level of detection of the assay, or a clonal evolution that can signify emergence of a treatment-resistant clone.

NGS of a panel of genes that could cover most mutations reported in MDS would be required for serial monitoring. However, including more genes in the panel for MRD would require more sequencing resources (total reads and read families) to meet the sensitivity and specificity requirements. Individualizing the design of a panel of selected genes that could be followed serially during treatment could be a solution. Such an approach is not implemented commonly in clinical practice, and there is the caveat that clonal evolution may not be detected.

(3) While complete remission and clearance of leukemia-specific genetic abnormalities are desired for AML to achieve long-term survival and diminish the risk of relapse, the treatment goal for MDS is largely variable depending on the patient’s overall performance, risk stratification, and the availability of tolerable therapies. In AML, patients that have favorable risk diseases could be monitored with MRD testing after completion of their intensive chemotherapy. Molecular relapse or concerning changes in the level of MRD may inform treatment decisions, such as instituting chemotherapy or performing alloHSCT. In contrast, in MDS patients receiving HMA without the prospect of alloHSCT, the treatment is continued as long as it is tolerated, as relapses/disease progression are expected. It should be noted that a minority of MDS patients may have prolonged responses to HMA treatment [39], though the mechanisms that lead to such a prolonged response, or the impact of achieving MRD negativity in the context of such a response, are not known.

(4) In AML, the blast percentage is usually higher in the bone marrow than in peripheral blood. Therefore, bone marrow from the first pull is the preferred sample for MRD testing [29]. However, for MDS, studies found a high concordance of the molecular genetic testing results between peripheral blood and bone marrow [40,41], although a study on the mutations of clonal hematopoiesis reported the possibility of localized clonal processes with slow dissemination to blood [42].

Studies have also demonstrated that the level of circulating tumor DNA (ctDNA), calculated from the concentration of cell-free DNA (cfDNA) and the mean VAF of mutations detected in cfDNA had high concordance with the mutation profile and VAF detected in the bone marrow [43]. The VAF in cfDNA may even more accurately reflect the bone marrow VAF [44,45]. Of interest, a study using cfDNA was able to detect cytogenetic evolution in a small number of MDS patients that did not respond to HMA therapy [46].

Therefore, easily accessible peripheral blood samples have the potential to be a reliable source for MRD detection in patients with MDS.

Other considerations using molecular techniques are the challenges that pre-existing clonal hemopoiesis and donor derived MDS, in the setting of alloHSCT, can pose. MRD detection based on mutation clearance in MDS is an attractive approach to post-transplantation monitoring to predict the risk of relapse. Rare cases of donor- derived MDS in post-HSCT patients have been reported [47,48], which may represent a challenge when interpreting MRD test results unless the molecular profile is distinct and non-overlapping from the pre-transplant setting.

In addition, CHIP mutations, particularly DTA mutations, are prevalent in older individuals. When present at low VAF, DTA mutations may or may not be associated with the pathobiology of the cytopenias of patients diagnosed with MDS [49,50]. Even though *ASXL1* mutations are known to be independently associated with poor prognosis in MDS and CMML (NCCN Guidelines for MDS, https://www.nccn.org/professionals/physician_gls/pdf/mds.pdf accessed on 3 Feburary 2024), DTA mutations are not specific to MDS. Additionally, DTA mutations are only highly predictive of myeloid neoplasms when coexisting with other mutations, such as those in *RUNX1*, *EZH2*, *CBL*, *TP53*, *NRAS*, *CUX1*, or *IDH2* [51]. It is possible that only subclones with co-mutations initiate the neoplastic process, and when the neoplastic subclone is cleared by treatment, the major CHIP clone can persist. For this reason, DTA mutations are excluded from NGS-based MRD testing for AML [29]. A study by Nannya et al. found that seven patients who achieved complete remission (CR) after azacitidine treatment had persistent major mutant clones with VAF > 0.40 [52]. The authors postulated that CHIP-related mutations persisted at a high VAF because these clones reverted to CHIP after treatment [52]. These results suggest that DTA mutations should also be excluded from NGS-based monitoring of treatment responses in MDS. However, these patients represented <10% of the 48 patients who achieved CR in that study [52]. Further studies are required to determine whether the reversion to CHIP is significantly associated with worse clinical outcomes. Another caveat of utilizing NGS/molecular assays is that germline mutations in the genes *ANKRD26*, *CEBPA*, *DDX41*, *ETV6*, *GATA2*, *RUNX1*, and *TP53* should be excluded as MRD markers [29].

There are many unanswered questions regarding the application of NGS for MRD detection in MDS patients. First, although most MDS patients harbor at least one genetic abnormality, the clinical significance of different mutations can be very different [53], as highlighted by the Molecular International Prognostic Scoring System for MDS (IPSS-M calculator https://mds-risk-model.com/, accessed on 3 February 2024) [54] and the NCCN Guidelines for MDS. More studies are also needed to investigate whether the dynamics of mutations of different clinical significance carry the same weight for MRD follow-ups. This will help to determine the best NGS panel for MDS MRD.

Second, although some publications have indicated that circulating tumor DNA (ctDNA) tests have the potential to replace testing on blood mononuclear cells (PBMNC), ctDNA tests that require a lower limit of detection may be more costly and technically challenging. For MRD detection in patients receiving treatment other than alloHSCT, there are insufficient data for conclusively suggesting the best detection sensitivity required for monitoring VAF changes to discriminate an effective treatment response from failed treatment. Finally, NGS-based MRD tests for MDS will require clinical and technical guidelines, and harmonization of wet-lab and uniform bioinformatics analysis and interlaboratory quality control protocols and proficiency tests will be required to standardize the test methods and protocols.

The most recent International Consensus Classification (ICC) [55] have introduced MDS/AML (cytopenic myeloid neoplasm with 10–19% blasts in the blood or bone marrow), as AML treatments can be utilized in such cases. The best approach for MRD testing of these patients is as yet unclear, although the principles of AML MRD testing should be applicable. Further studies are required to determine whether all mutations have the same value in predicting relapse and long-term survival in MDS/AML, similar to that observed in AML.

MDS exhibits not only DNA-based genetic abnormalities that could be detected by NGS, but also heterogenous aberrations related to the immunophenotype, gene expression, epigenetics, microenvironment, and immune response. Therefore, utilizing single cell sequencing (SCS) to investigate the broad aspects of MDS appears to have promising potential [56]. A significant advancement in the high-throughput multi-omics SCS has been achieved in recent years as an expected natural evolution following the development of cytogenetic and molecular technologies designed to characterize the clonal propagation of hematologic neoplasms [57]. Microfluidics/microdroplets, plate based, nanowells, and fluorescence activated cell sorting (FACS) are high-throughput (>5 K cells) SCS technologies [58,59] that have been applied to study the tumor’s transcriptome via single-cell-RNA-sequencing (scRNA-seq), the epigenome via scEpigenomics (e.g., ATAC-seq), and the genome aberrations via scDNA-seq. Although this technology has been utilized for a variety of cancers, hematologic neoplasms such as MDS and AML could be most suitable for these applications considering the inherent state of blood and bone marrow aspirate samples being in natural cell-suspension. SCS technologies applications in MDS and AML could interrogate these neoplasms at the single cell level to elucidate intra-tumor heterogeneity, clonal architectures (clonal phylogenies and trajectories), pre-MDS or pre-AML stem cells, identifying clonal hematopoiesis (CH)/CHIP, and, most interestingly, assessing MRD [58,59,60,61,62]. In addition, the unique ability of SCS technology for studying the proteogenomic makeup of individual cells via sequencing the genomic DNA and the oligonucleotide-labeled conjugated antibodies allows for simultaneous characterizing of the cell genotypic and immunophenotypic features [63] in contrast to inferring such by traditional MFC and bulk NGS. Coupling of MFC and bulk NGS by correlating their findings infers speculatively about the immunophenotypic and genetic characteristics of dominant leukemic population (blasts) in AML; however, it becomes challenging when dealing with less dominant blast population (low blast count) or heterogenous samples, such as those from patients with MDS. To the best of our knowledge, only two scientific studies applied the single cell technology for the assessment of MRD in AML [61,64]. Ediriwickrema et al. studied 15 patients with AML at different timepoints of diagnosis, remission, and relapse. The study by Robinson et al. analyzed 30 post-induction chemotherapy samples from AML patients by single cell-MRD (sc-MRD) assay (proteogenomic SCS) with similar sensitivity after initial enrichment of CD34^+^ and/or CD117^+^ progenitors using FACS, multiplexing several patients’ samples per run by identifying patient-specific single nucleotide polymorphisms (SNPs) to aid the “demultiplexing of sc-MRD sequencing data.” Both studies delineated the dynamics of detected neoplastic clones and identified the mutations representing CH and those associated with the relapsed disease with the future prospective of potential utilizing the SCS for the monitoring of clonal fate and evolution during therapy. However, SCS could be limited by low single cell throughput, low number of recovered cells available for sequencing at the end of the analytical process, small DNA panel size, and allele drop out [61,62].

Considering that SCS is principally shown to be capable of detecting low levels of residual disease (single cell-MRD) and providing additional information beyond the mere MRD usually assessed by traditional methods, this technology could be theoretically applied to MDS aiming to (1) assess the dynamics of low levels of disease and representative clones/subclones and dissect the genomic makeup of the disease at the single cell level, (2) couple the immunophenotypic and genetic information to elucidate the clonal hierarchy of affected lineages that could be patient specific, (3) monitor the dynamics of the neoplastic clones/subclones as altered by therapeutic modalities, (4) anticipate the emergence of resistant clones, and (5) identify proteogenomic patterns that may predict response or resistance to certain therapy and correlate with count recovery or transfusion independence via prospective clinical trials. As the assessment of MRD in MDS has not been standardized and there are no specific recommendations regarding limit of detection [12,65], sc-MRD with a reported sensitivity of 0.1% could be theoretically applicable, especially in patients with low-risk disease (blast count is usually <1–4%), as there is no available curative therapeutic modality outside alloHSCT. In contrast, for patients with higher-risk MDS with increased blasts (typically blast count is 5–19%), conventional MRD-assays could be utilized.

In addition, although sc-MRD has not been yet explored in MDS, SCS technologies have already been utilized to study other aspects of this neoplasm, such as the intra-tumor heterogeneity of MDS with isolated del5q elucidating different clonal architectures in different patients [66] and the MDS stem cells by sc-RNA-seq elucidating the transcriptional profile of these cells [67]. Considering the multifaceted pathogenesis and oncogenesis of MDS, studying MDS merely by sc-DNA-seq may limit our understanding to only one aspect of this neoplasm; it would be intriguing to comprehensively investigate other aspects of transcriptomic, epigenomic, and tumor microenvironments at predetermined timepoints within a prospective clinical study.

## 3. Cytogenomic-Based Detection of MRD in MDS

Cytogenetic evaluation has played a major role in determining clonality and has been an essential parameter in the diagnosis of MDS [4]. Chromosomal abnormalities are observed in 50–60% of patients with MDS. The most frequent single cytogenetic abnormalities include del(5q), monosomy 7 or del(7q), trisomy 8, and del(20q) [68,69]. In addition to establishing a clonal process in patients with peripheral blood cytopenia, chromosome analysis, fluorescence in situ hybridization (FISH), chromosomal microarray, and mutational testing by NGS platforms provide a comprehensive step-wise approach in prognostication and clinical-morphologic correlation of MDS, therapeutic strategies, and in predicting the likelihood of progression to AML. Unlike many other hematologic malignancies with a single cytogenetic defining event in diagnosis, i.e., chronic myeloid leukemia and acute promyelocytic leukemia, there are various combinations of chromosomal abnormalities that are associated with MDS. According to the World Health Organization (WHO) Classification, MDS may be diagnosed by MDS-defining cytogenetic abnormalities in patients with unexplained cytopenia, such as considering MDS with low blasts and isolated 5q deletion as a unique MDS entity [4].

Results from cytogenetic studies, either by conventional karyotyping (G-banding), FISH, or chromosomal microarray, are included in the comprehensive cytogenetic scoring system (CCSS) adapted by the WHO, which defines the specific prognostic stratification of MDS based on existing cytogenetic abnormalities [68]. MDS demonstrates a higher prevalence of unbalanced chromosomal abnormalities in contrast to AML, which are usually characterized by balanced structural abnormalities involving translocations and inversions. The genetic aberrations in MDS are typically chromosomal loss of genetic material, such as deletions and monosomies, while gain of genetic material (i.e., trisomies) and, to a lesser extent, balanced rearrangements [69] are also encountered.

The CCSS has been incorporated into the revised International Prognostic Scoring System (IPSS-R) score [70] and, more recently, the IPSS-M [54] for MDS, thereby allowing the prediction of clinical outcomes for MDS patients and potentially assisting in the design of clinical trials for disease. Both the IPSS-R and IPSS-M encompass the five cytogenetic subgroups as described in the CCSS and account for chromosomal aberrations in the overall score values for MDS [70].

Genetic heterogeneity involving recurrent chromosomal abnormalities of MDS has been well documented [69]. Cytogenetic assays that globally purview the genome, such as routine chromosome analysis (karyotype G-banding) by evaluating twenty metaphases or chromosomal microarray, are utilized for the evaluation of MDS. FISH may complement conventional cytogenetic analysis in situations of failure of standard G-banding (absent or poor-quality metaphases). FISH analysis of del(5q) or with del(7q) or monosomy 7 may provide prognostic information [71,72]. Targeted FISH for deletion 5q may be indicated for testing, as it is a recognized WHO classification entity of MDS with low blasts and isolated 5q deletion. Although FISH is specific, with limited sensitivity, it is important to recognize that FISH can only be applied in a targeted fashion; hence, comprehensive assessment for chromosomal aberrations cannot be carried out using this technique due to the spectrum of recurrent chromosome abnormalities for MDS. However, FISH may be useful for clarifying complex aberrations, and it can detect abnormalities in up to 15% of karyotypically normal MDS patients [71,72,73].

Single nucleotide polymorphism (SNP) chromosomal microarray (CMA) is an important tool in identifying chromosomal imbalances and loss of heterozygosity (LOH) that are not detected by standard cytogenetics methodology [74,75,76]. SNP-CMA is a feasible technique available to identify copy neutral loss of heterozygosity (CN-LOH), a form of allelic imbalance in which a heterozygous region of the chromosome becomes homozygous due to uniparental disomy. SNP-CMA is useful in myeloid neoplasms with insufficient metaphases (<20) or failed karyotypes due to poor quality of the specimen or due to factors inherent to disease, such as bone marrow failure. For such MDS patients with karyotype showing very good, good, or intermediate cytogenetics IPSS-R groups, SNP-CMA might be helpful to further assist with diagnostic uncertainty and risk stratification if additional copy number alterations (CNA) or LOH abnormalities are ascertained.

Kanagal-Shamanna et al. highlighted recurrent CNA and CN-LOH for diagnostic evaluation and assessment of prognosis of MDS and MDS/MPN [77]. In the setting of normal karyotypes, CN-LOH/LOH serves as a clonal marker in MDS. Regions of CN-LOH may have a similar diagnostic significance equivalent to a loss of the homologous allele on the other chromosome (i.e., deletion 7q is similar to 7q LOH). Identification of large CN-LOH should indicate additional mutational analysis of target genes of potentially predictive or therapeutic significance, such as a CN-LOH of chromosome 9p containing a homozygous V617F mutation [78].

The combination of metaphase cytogenetics, FISH and CMA led to a higher diagnostic yield of chromosomal defects compared to that detected by metaphase cytogenetics alone, often through detection of novel lesions in patients with normal or non-informative karyotype results [76]. Although response to lenalidomide among MDS patients with del(5q) and tyrosine kinase inhibitor (TKI) resistance among chronic myeloid leukemia (CML) patients did not correlate with CMA findings, additional copy number aberrations identified by CMA and TP53 mutations/17p deletions are associated with disease progression and worse prognosis [79,80]. Although CMA has many advantages, the assessment of genomic aberrations by CMA testing is not widespread in MDS; NCCN guidelines for MDS support CMA testing if a karyotype cannot be obtained and as consideration for patients with normal cytogenetics (NCCN Guidelines for MDS, https://www.nccn.org/professionals/physician_gls/pdf/mds.pdf accessed on 3 Feburary 2024). Moreover, the European LeukaemiaNet 2013 has suggested the use of CMA testing for the diagnosis of primary MDS [81]. Evidence based support for and suggestions for clinical utilization and methodology consideration for CMA have also been proposed [77].

More recently, optical genome mapping (OGM) has emerged as a novel single-platform cytogenomic technique that enables high-throughput, accurate and genome-wide detection of copy number variants (losses/gains) and structural variants (inversions, balanced and unbalanced fusions/translocations), which are important for diagnosis and risk-stratification of MDS. Given its ability to detect these abnormalities at high resolution, OGM is far superior to current standard-of-care cytogenetic techniques that include conventional karyotyping, FISH, and chromosomal microarrays. Several studies have demonstrated the validity the clinical utility of OGM in MDS, as many of these studies revealed additional prognostic information compared to conventional cytogenetics [82,83,84,85,86]. Moreover, Yang et al. demonstrated that 51% of the structural variants seen in the patient cohort were cryptic aberrations of prognostic and therapeutic significance, such as rearrangements involving *MECOM*, *NUP98::PRRX2,* and *KMT2A* partial tandem duplication [83]. These cryptic variants obtained by OGM provided additional information to change the IPSS-R risk scores [83]. More importantly, aberrations identified by OGM may provide markers for MRD monitoring for MDS.

The clinical utility of the cytogenetic methods for detection of MRD in MDS has been more elusive due to the inherent limited sensitivity of the cytogenetic assays. It is well known that routine cytogenetic analyses have low sensitivity in regard to the detection of residual neoplasia considering that, for chromosomes, only 20–30 metaphases are analyzed, for SNP-CMA at approximately 20% sensitivity, and targeted FISH testing at validated sensitivity levels established independently by different laboratories. Routine cytogenetic analysis via chromosome (G-) banding and FISH are standard procedures at the time of diagnosis and can be used for following up residual disease detectable by these assays. Appearance of the same abnormality during treatment or during relapse could be considered a measurable parameter of residual disease. Clonal cytogenetic evolution, such as acquiring new chromosomal aberrations over time, is relevant for the progression of MDS. In patients with IPSS-defined lower-risk MDS, acquisition of cytogenetic abnormalities is associated with poor prognosis and transformation to AML [87]. Therefore, sequential cytogenetic analysis of follow-up samples has been important in identifying the cytogenetic response, especially for high-risk MDS [12] and monitoring for clonal evolution, leading to greater understanding of the heterogenous acquisition or loss of genetic events in MDS [88]. Baseline FISH studies at primary diagnosis for the key clonal abnormality based on the karyotype or CMA findings could be considered to customize an informative probe/s for future monitoring of residual disease post therapy. Furthermore, an abnormal karyotype at the baseline appears to predispose toward the acquisition of further cytogenetic alterations, although no specific patterns of clonal evolution emerged according to the baseline karyotypic anomalies [89].

The cytogenomic studies of MDS can be evaluated at different time points, including after completion of chemotherapy, after-post remission therapy, and before and after alloHSCT. Detection of residual disease by cytogenetic analysis in MDS is based on the premise that malignant myeloid cells divide readily in culture, thereby allowing exploitation of routine cytogenetics to identify clonally abnormal cells. Possible alternate approaches to increase sensitivity of detection of residual disease in MDS could be achieved by performing FISH or SNP-CMA on lineage specific CD34^+^ (or alternatively, CD117^+^) enriched cells from peripheral blood or bone marrow after magnetic cell separation or flow cytometric sorting [90,91]. Drawing potential parallels, in patients with MDS/AML in CR after alloHSCT, Stasik et al. demonstrated a high sensitivity of 100% and specificity of 91% for detecting molecular relapse by NGS [90]. In similar approaches in multiple myeloma, immune-magnetic CD138-positive cell sorting plasma cells significantly increased the percentage of abnormal cells identified in FISH analysis [92].

In patients with complete cytogenetic response, Tehranchi et al. demonstrated that the 5q deletion remained detectable in patients with MDS-del(5q), using FISH of sorted CD34^+^, CD38^−^/low, and CD90^+^ HSCs at the time of complete response during lenalidomide treatment [93], further indicating that complete cytogenetic response does not necessarily reflect and capture the quiescent malignant stem cell population. In these situations, MRD testing in selected cells will indicate whether malignant stem cells are eradicated by therapy. Such immunotypic enrichment allows for selective characterization of MRD using targeted FISH for the original clonal abnormality and/or genome-wide assessment for clonal evolution using SNP-MA or OGM (copy number and structural variants) for MDS.

Positive cytogenetic MRD by any of these assays, provides valuable information regarding therapy response, relapse, and prognosis. AML patients with cytogenetically adverse-risk disease at diagnosis are more likely to have persistent abnormal cytogenetics [94]. In some studies, detection of abnormalities in submicroscopic number of AML cells has prognostic significance in the context of alloHSCT [95,96,97,98,99,100]. In most instances, detecting residual AML by cytogenetics in patients who underwent alloHSCT predicted early relapse and shortened survival [100,101]. The presence of cytogenetic adverse-risk group subclones at the time of diagnosis in patients with MDS has an unfavorable influence on survival and AML progression [98], and MRD of these cytogenetic abnormalities could predict relapse or resistance to therapy by sequential cytogenetic monitoring.

## 4. Multiparameter Flow Cytometry-Based Detection of MRD in MDS

**Figure 2:**
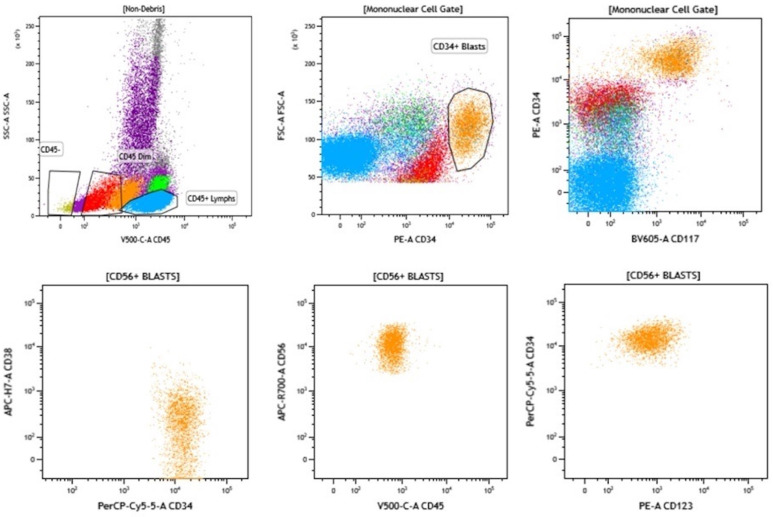
A CD34^+^/CD45^dim^/CD56^+^/CD38^dim/−^ population of aberrant myeloblasts in MDS.

As a fast and reliable testing tool, multiparameter flow cytometry (MFC) offers a variety of qualitative and quantitative analyses and characterization of hematopoietic cells of all lineages in MDS. In recent years, the use of MFC has provided a non-invasive and quantitative method for monitoring MRD in acute myeloid leukemia [102] and MDS patients undergoing treatment; additionally, in the post-alloHSCT setting [103], MFC allows for the identification and characterization of hematopoietic cell lines and the detection of aberrant expression patterns. The European LeukemiaNet Minimal Residual Disease Working Party recommended applying a comprehensive panel, including progenitor cell markers (CD34, CD117), myeloid and monocytic lineage markers, and differentiation antigens (CD2, CD7, CD19, or CD56), as well as using a different-from-normal (DfN) approach and automated data analysis testing for optimization of MFC to establish MRD in AML [29,102]. Distinct expression profiles with features of aberrancy can be indicative of MDS, including decreased neutrophil granularity (decreased SSC ratio), increased immature progenitor cells with expression of CD34, HLA-DR, and/or CD117, bright CD36 in CD45 dim myeloblasts, increased frequency of CD38 dim or negativity or increased CD123 expression in myeloblasts, aberrant CD56 expression in myeloid and monocytic lineage cells, decreased CD15 expression in maturing myeloid cells, and aberrant CD5, CD7, and CD19 expression, decreased or absent CD10 expression despite bright CD15, and decreased CD33 expression in myeloid cells [104,105,106,107,108,109,110] (Figure 2). Such aberrancies in progenitor and differentiated hematopoietic stem cells can be detected and quantified by MFC, with a limit of detection (LOD) ranging from 0.1% to 0.01% [111,112]. In addition, FC enables analysis of dysplastic changes in other rare hematopoietic cells, including basophils, eosinophils, and mast cells in MDS [113,114,115].

There are two commonly used MFC analyses: (1) The leukemia-associated aberrant immune-phenotype (LAIP) approach, which is based on the assumption that residual disease retains the same phenotypic aberrancies of the initial disease, and the (2) “difference from normal” approach, which is designed to analyze immunophenotypic aberrancies regardless of the initial disease [116,117,118]. Both approaches harbor limitations, including reduced sensitivity and specificity due to clonal involution and immunophenotypic switches in dysplastic cells, particularly in the LAIP approach, and heterogeneity in the pre-analytical and analysis stages.

According to recent studies, assays designed to detect leukemia stem cells by MFC increase the sensitivity and predictive value [119,120,121,122]. Van Rhenen et al. showed that the CD34^+^CD38^−^ population in AML and high-risk MDS samples at diagnosis is resistant to chemotherapy, and detection of this population using FACS assay can be used for monitoring the disease and characterization of chemotherapy-resistant neoplastic cells [119].

The Euroflow Consortium has introduced a high-throughput eight-color EF assay for AML/MDS to detect neoplastic blasts and characterize the lineage [123,124]. However, similar to other MFC assays, this panel’s utility in different cytogenetic groups and differentiation of normal hematopoietic stem cells from neoplastic blasts is not well documented [125].

Despite recent evidence supporting the diagnostic utility of MFC detection of MRD in MDS, a consensus of universal standardization of practice has yet to be reached regarding sample types to analyze, the technical analysis process, gating strategies, and the data interpretation. The European LeukemiaNet has recently provided pre-analytical and technical recommendations to standardize the MRD analysis [126]. The group recommends using bone marrow samples aspirated into heparin tubes, ideally processing the samples within 24 h, and application of a comprehensive antibody panel that includes immaturity markers (CD34, HLA-DR, CD117), myeloid and monocytic markers, and differentiation markers for cross-expression. Recent studies have investigated the utility of artificial intelligence using supervised machine learning for MFC data analysis in AML and MDS [118,127,128,129,130]; however, this technique is still in its infancy and larger scale case data is warranted. Further validation studies, including MDS with cytogenetic variations, are warranted to establish a universally standardized approach in detecting minimal residual disease in MDS.

## 5. MRD Monitoring Prior to alloHSCT or in the Non-alloHSCT Setting

In other hematologic malignancies, utilization of MRD analysis has recently contributed to the optimization of therapy [131].

MDS is a clonal malignancy, and hypomethylating agents (HMA) do not eliminate the abnormal clones even if CR is achieved as relapses inevitably occur. Even in patients that underwent alloHSCT late, relapses can occur and are the result of quiescent MDS stem cells [132]. Several studies in the context of MDS have reported MRD using different techniques in the setting of different MDS-directed therapies (Table 1). Moreover, some studies comprised a heterogeneous population, including a preponderance of patients with AML, so it is often challenging to determine if these results are entirely applicable to patients with MDS.

The Hovon-SAKK AML trials also encompassed patients with high risk MDS [133]. An analysis from multiple studies included patients with an IPSS score of at least 1.5 or R-IPSS > 4.5 (depending on the included study) that were treated with intensive chemotherapy. MRD analysis by MFC revealed that MDS patients that received a second induction chemotherapy had a trend of higher MRD positivity compared to AML patients. The analysis included 73 MDS patients versus 1064 AML patients. The MRD positivity impacted OS. The study population was re-analyzed based on the ICC new classification encompassing also the MDS/AML category. Included in this MRD analysis were patients who received two cycles of induction, achieved CR/CRi and had available bone marrow samples for analysis. Patients with MDS had a trend for higher MRD positivity (33%) compared to AML or MDS/AML (22% and 18%, respectively). MDS patients with MRD positivity had worse outcomes compared to MRD negative MDS patients, and the three-year OS for MRD positivity was 18%. Notably, patients with MDS in this study had, by definition, less than 10% blasts, and the cytogenetics were reported to fall into adverse risk. The study did not report on mutational status or the presence of *TP53* deletion [133].

In a study utilizing intensive chemotherapy and pravastatin for AML and MDS, two patients with MDS enrolled [134]. Both achieved CR without MRD by MFC. One patient relapsed; notably only 4 out of 12 patients with CR with MRD negativity relapsed in the median interval of 4 months. The study did not provide detailed information regarding the MDS patients, although one of the two proceeded to alloHSCT [134].

An important study in the context of MRD and MDS was recently reported. The Stimulus-MDS1 study included patients with high risk MDS that were randomized to Sabatolimab or placebo, with all patients receiving HMA chemotherapy [41]. This study utilized an NGS-error-corrected panel. For the MRD analysis, *DNMT3A*, *TET2* and *ASXL1* mutations were excluded. The study reported high concordance between peripheral and bone marrow samples’ regarding variant detection. Samples from 106 patients were analyzed for MRD analysis by NGS (56 patients on Sabatolimab arm and 50 on placebo arm). The MRD cut off clearance was 0.2%, 0.5% and 1%. Over 80% of patients attaining MRD negativity irrespective of cut off level achieved by CR/marrow CR. Patients on sabatolimab arm attained MRD-negativity in higher proportion compared to placebo (for the 0.2% cutoff 16 vs. 6%, respectively and for 1% cut off 35.7% vs. 18%). Patients with MRD-negativity even at the cut-off of 1% had better outcomes compared to those that remained MRD positive pertaining to OS and PFS [41]. It is of interest that the sabatolimab arm had a longer duration of response compared to placebo, and the MRD data are intriguing in regard to a possible explanation.

In the pre-transplant setting, the role of MRD in MDS is being explored. Although it is accepted that the blast percentage (such as 10% cut off) impacts post-transplant outcomes, the necessity of achieving CR has been debated. A recent retrospective study from China utilizing MRD by MFC explored the role of MRD in the pre-transplant setting. The study examined results of 103 patients with MDS with excess blasts treated with various chemotherapy regimens and achieved CR (and had MRD data available) prior to alloHSCT [135]. The MRD MFC was based on a multicolor assay (initially 4-color before including up to 10-color over time), with sensitivity ranging from 0.05% to 0.01%. Thirty-six patients were MRD positive and 67 patients were MRD negative. The 3-year DFS for patients with MRD negativity was 85% vs. 73% for those that were MRD positive (*p* = 0.049). In both univariate and multivariate analysis, the detrimental impact of MRD positivity impact on DFS was retained. Similarly, for 3-year OS MRD, negativity was associated with 91% (vs. 70% for MRD positivity) probability of survival. The three-year cumulative relapse rate was more than four times higher in the MRD-positive (16% vs. 3.4%) cohort [135]. Only the status of MRD impacted outcomes in both DFS and OS analyses. However, the study did not include associations with mutational data, and only ~29% patients were treated with HMA [135]. The latter likely is related to the fact that the study also included a younger population (median age 41 years- old) that can tolerate intensive chemotherapy. The mechanisms of relapse in the setting of MRD -positivity may include re-emergence of chemo-resistant clones, but this remains speculative, as confirmatory data in the post-alloHSCT setting was not available. Another limitation of this study is that molecular risk stratification, hematopoietic cell transplantation-specific comorbidity index, and conditioning intensity were not provided.

A study of WT1 gene expression demonstrated that in childhood MDS patients there is a potential use for MRD monitoring from peripheral blood samples given differences in expression compared to healthy volunteer samples [136]. In a metanalysis, 450 patients with MDS were included [137]. The analysis included two studies that examined the WT1 as an MRD marker post alloHSCT. The WT1 overexpression was associated with a higher risk of relapse [137]. The authors of study cautioned regarding the role of pre-alloHSCT levels of WT1, as there is no clear consensus.

Eprenetapopt (APR-246) was combined with Azacitidine (AZA) in Patients with *TP53*-mutated MDS and “Oligoblastic” AML as part of Phase II clinical trials [138,139,140]. The study by Sallman and colleagues enrolled 40 patients with MDS. The median VAF for *TP53* was 20% and the range was 1 to 72%; the overwhelming majority of patients had complex karyotype. Fifty percent of MDS patients achieved CR, with 38% achieving complete cytogenetic remission. The NGS MRD negativity (set at a value of <5% VAF for *TP53*) was achieved in 43% of patients. Forty three percent of patients proceeded to alloHSCT. Patients with NGS MRD negativity had a trend toward improved survival compared with those with MRD positivity; however, the small number of patients precludes drawing firm conclusions.

In a similar study performed in France, patients with MDS were enrolled in the combination of azacitidine and Eprenetapopt [138]. Thirty-eight patients with MDS were enrolled. The majority had high and very high R-IPSS; the *TP53* median VAF was 20 (range 0.1–83%). Focusing on MDS patients, forty seven percent reached CR; the median OS was 12 months. The study did not differentiate between AML and MDS patients when reporting *TP53* VAF results; 73% of patients that responded achieved MRD negativity, while a sizeable percentage reached VAF less than 0.1%. Attaining MRD negativity (VAF less than 5%) was associated with statistically significant improved outcomes, including OS and duration of response [138]. MRD-negativity was associated with an almost three-fold increase in median OS compared to patients that were MRD positive (median OS 5 months) [138]. In an abstract presented at the American Society of hematology (ASH), the results of both studies were combined [139]. Overall, the studies combined included 74 patients with MDS. The CR/PR rate for MDS patients was 49% and the overall response rate 70%. The abstract presented at ASH did not report separate outcomes for MRD analyses by NGS between different subgroups [139]. Overall, MRD negativity for *TP53* (5% VAF cut off) was noted in 40 patients; MRD negativity was associated with responses that were mostly CR/PR [139]. MRD-negative patients had a two-year overall survival of 50%; which was more than double that of the MRD-positive patients, although this was not statistically significant. Importantly, patients with NGS MRD-negativity or CR/PR that underwent alloHSCT had a superior median OS compared to patients that have not attained this disease status prior to alloHSCT [139].

A seminal study was the phase III randomized clinical trial Blood and Marrow Transplant Clinical Trials Network (BMT CTN) 0901 [141]. In this study, 54 patients with MDS were randomly assigned to myeloablative conditioning (MAC) or reduced intensity conditioning (RIC) regimens. The study called for blasts to be less than 5% prior to alloHSCT, and for 48 patients it was possible to assess mutational status prior to the conditioning regimen [141]. Forty two percent of patients had a detectable mutation when 10 genes were tested (*FLT3*, *IDH1*, *IDH2*, *JAK2*, *KIT*, *NPM1*, *NRAS*, *RUNX1*, *SF3B1*, and *TP53*) prior to alloHSCT with a median VAF of 0.7%. The number of variants ranged from 1 to 11, with a median number of 2. Those with detectable mutations prior to alloHSCT were found to have increased rates of 3-year relapse (40% vs. 11%) and decreased OS (55% vs. 79%). Those with detectable mutations had lower relapse free survival in RIC compared to MAC arms (13% vs. 49%).

Magrolimab is a monoclonal antibody targeting CD47 epitope [142]. It acts as macrophage checkpoint inhibitor, allowing the macrophages to attack leukemic cells. Magrolimab was used in several clinical trials. In the context of MDS, results regarding MRD were reported in early phase clinical trials [143]. In this clinical trial, azacitidine was used in conjunction with Magrolimab in patients with higher risk MDS. Enrollment criteria required at least intermediate risk by R-IPSS, and 95 patients were treated. The majority of patients had poor or very poor cytogenetics by R-IPSS, and 26% had *TP53* mutations. Thirty-two percent of patients achieved CR; 41% of these patients achieved MRD-negativity by MFC. The median duration of follow up for survival was 17 months. Patients that attained MRD negativity appeared to have a trend for better outcomes compared to those with MRD positivity. Twenty percent of patients that underwent alloHSCT were MRD negative; median OS was not reached.

A study from Dana Farber examined the impact of mutations in the context of MDS and alloHSCT [144]. The study investigated several parameters including blast percentage (using 5% as cut off) prior to alloHSCT. A strength of the study was that the analysis was performed shortly before alloHSCT (median of 9 days). In the multivariate model, mutations in *TP53* and *TET2* were associated with a detrimental impact in the OS [144].

In another study, samples of patients with MDS that underwent alloHSCT were sequenced at a depth of 1500×, and the cut off for variant allele frequency was set to 10% [145]. In a multivariate analysis, including blast percentage (cut off 5%) at time of AlloHSCT, the presence of *TP53* and *IDH2* was associated with decreased 3-year OS [145]. The study [145] utilized samples that were collected within a month prior to alloHSCT. Both studies [144,145] included mutational analysis but did not specifically address MRD and did not report the total number of patients that were in CR at time of alloHSCT.

A study included 449 patients with MDS, MDS/MPN overlap and oligoblastic AML treated with azacitidine; pre- and post-treatment NGS mutation profiles were collected [52]. Patients were treated in Sweden or Japan, and the majority (n:384) had MDS. For the whole patient population (but less pronounced for the MDS/MPN subgroup), a decreased major clone size correlated with response based on IWG criteria. In the 48 cases of CR, the median post treatment VAF was as low as 0.066 compared to 0.77 and 0.84 for stable and progressive disease, respectively. Furthermore, 35 patients in CR or marrow CR achieved complete molecular remission (defined as less than 1% of major driver mutated clones). However, in some cases DTA harboring clones persisted, highlighting the complexity of factoring clonal hemopoiesis when utilizing NGS techniques. Another finding was that patients with germline *DDX41* mutation could have a poor response, even though the post treatment clone size was small. Despite comprehensive investigation, the mechanisms behind this finding remain unclear. The size of clone post treatment had impact in OS. The study utilized the post treatment maximum VAF to design the prognostic scoring system after azacitidine treatment (PSS-AZA). Four patients harboring *TP53* mutations that underwent alloHSCT and had a decrease in clone to less than 0.1 remained in CR or molecular CR after a median follow up of 2 years.

Testing ctDNA using serial post-treatment blood samples confirmed the decrease in ctDNA levels after effective treatment [43,146] and the dynamics of ctDNA mutation profile and VAF provided accurate information on the response of neoplastic clones and subclonal evolution [146]. The study by Zhou et al. [43] included 21 patients with MDS but included also patients with AML. The majority of MDS patients received HMA. The post-treatment ctDNA was lower in patients that achieved response; ctDNA positivity was associated with a shorter PFS (median 5.6 months) and OS (median 11 months). An increase in the mean ctDNA VAF predicted decreased PFS. The emergence of either previously detected or new mutations harbingered a recurrence [43]. The study did not specifically report outcomes of patients with MDS that had response and ctDNA positivity/undetectable levels. The study of Yeh et al. included 12 patients with MDS (with the majority having at least intermediate risk by R-IPSS) that received azacitidine and eltrombopag [146]. The study reported an excellent correlation between mutation detection in bone marrow and ctDNA, even in cases of leukopenia [146]. The study reported that, even in patients with response to treatment, the ctDNA monitoring was able to detect new mutations that became dominant clones and ctDNA changes that heralded disease progression [146].

A large cohort of MDS (n:247) patients that was followed by NGS was reported from the Moffitt Cancer Center and Research institute [147]. Approximately 80% of patients had at least an intermediate risk by R-IPSS and were treated with HMA. Eighty-six percent of the patients had at least one mutation. The study used a VAF of 5% for SNV and 10% for insertions/deletions. Sequential monitoring was performed in 108 patients. Clearance of mutations was not associated with improved OS, apart from *TP53* (15.6 months vs. ~8 months when *TP53* mutation persisted). Although VAF was similar to other studies reporting MRD, the authors did not use MRD terminology in their study [147].

Festuccia et al. [148] investigated the role of MRD and different conditioning regimens. In their study the term Minimal Identifiable Disease (MID) for patients that had less than 5% blasts in bone marrow. The study utilized MFC [sensitivity range from 0.1% to 0.001%] and cytogenetics [chromosome and FISH analysis]; study defined MID positivity for presence of cytogenetic abnormalities and MFC positivity [148]. Twenty-two percent of patients were MID negative at alloHSCT; 53% were MID positive. The study included 289 patients that were transplanted between 2004 and 2013 to determine MRD. The majority of patients had de novo MDS and 28% had secondary MDS. Conditioning regimens were grouped as high or low intensity; majority of patients received the former. The MID positivity by cytogenetics, had a detrimental impact for patients that received low intensity conditioning.

In the study of Craddock et al. [149] patients with MDS or AML were randomized in two arms [Fludarabine based RIC or experimental arm (FLAMSA-Bu)]. The study included 80 patients with MDS, and MRD was based on MFC (limit of detection 0.02–0.05%) prior to alloHSCT and at day +42. The authors used different methodologies for MFC MRD [149]; when stringent criteria were applied (in most cases of conventional MRD corresponding to at least 0.2%), MRD-positive patients had a two-year cumulative incidence of relapse (CIR) 50% vs. 20% for MRD-negative. The study did not report outcomes separately for MDS patients.

## 6. Peri-Transplant MRD Monitoring in Patients with MDS Undergoing Allogeneic Hematopoietic Stem Cell Transplantation (alloHSCT)

MFC (with at least 10^−4^ sensitivity) was employed to assess MRD both before conditioning chemotherapy and at day +100 in patients with AML or MDS undergoing alloHSCT [150]. Reduced intensity conditioning was utilized in most patients included in this analysis. Seven of the 38 patients with MDS ultimately experienced post-transplant relapse. Although analysis was not stratified according to underlying hematologic malignancy, MRD-positivity at day 100 following alloHSCT was associated with increased cumulative incidence of relapse at 2 years (53% compared to 11%, *p* = 0.043) and decreased overall survival (53% compared to 82%, *p* = 0.043). Event free survival at 2 years was 40% vs. 76% for patients with MRD-positive and MRD-negative state post-transplant, respectively. In contrast, neither mixed CD3 chimerism nor pre-transplant MRD-positivity significantly impacted relapse risk in this cohort. Notably, in a logistic regression model, pre-transplant MRD was associated with positive post-transplant MRD.

A single-center study assessed MRD in 78 high-risk or very high-risk patients with MDS in the post-transplant period [151]. MRD was monitored in bone marrow samples by both four-color MFC and WT1 PCR at regular intervals. MRD-positivity was identified in 21 patients, and this corresponded to an increased cumulative incidence of relapse at 2 years after transplantation by both WT1 (*p* = 0.040) and multiparameter flow cytometry (*p* < 0.001) monitoring. The 2-year relapse rate was only 27.3% in MRD-positive patients in this study (compared to 4.5% in MRD negative patients, *p* = 0.003), although longer-term outcomes have yet to be reported. However, recent MRD guidelines in AML have recommended leukemia-specific PCR assays over less specific WT1 expression. Additionally, peripheral blood assessment of WT1 PCR is preferred over bone marrow analysis due to higher background levels of WT1 expression in normal bone marrow [29].

Another study utilized enhanced exome sequencing and identified somatic mutations in 65 patients with MDS undergoing alloHSCT [152]. The risks of disease progression and death were significantly higher in patients with at least one mutation detected at a VAF of 0.5% in bone marrow samples on day 30 post-transplant [152]. Similarly, MRD-positivity on day 100 after transplant was also associated with worse progression-free survival. Yun et al. [153] also monitored MRD by NGS including 37 genes in both the pre- and post-transplantation setting. MRD-positivity was defined as VAF > 5%. In 37 patients with MDS, CMML, or secondary AML, post-transplantation NGS-negativity was associated with significantly improved overall survival.

In a study including 14 patients with MDS who underwent myeloablative conditioning, post-transplant MRD was quantified by personalized droplet digital PCR (ddPCR) with a median detection limit of 0.04% [154]. In addition, ctDNA was also analyzed, and those patients with persistent ctDNA at either 1- or 3 months post-transplant had a higher risk of relapse. However, these results were not stratified by underlying hematologic malignancy (MDS compared to AML).

Another prospective study recently investigated individualized MRD assessment utilizing ddPCR [37]. Study involved patients with MDS and related neoplasms (e.g., AML, Chronic Myelomonocytic Leukemia, MDS/MPN-U, therapy related myeloproliferative neoplasms) that underwent alloHSCT. From the 177 MDS patients, the majority were MDS-EB1 (n:52) and MDS-EB2 (n:62). The study did not report in different subgroups and 61 patients relapsed after alloHSCT. Bone marrow MRD-positivity preceded clinical relapse in 42/54 patients by a median of 71 days [37]. Notably, 28 patients with MRD-positivity and relapse had comparison of peripheral blood and bone marrow samples. In 8 of the 28 patients, only the BM sample was MRD positive prior to relapse. Using a cut-off of 0.1% VAF the 1-year relapse after MRD positivity was 41%, with an RFS of 49%, respectively. Patients with decreasing MRD had an RFS comparted to those with stable or increasing MRD level. Although MDS patients were the majority of the cohort, conclusions are limited due to inclusion of other myeloid malignancies [37].

A retrospective study included 115 patients with MDS undergoing a myeloablative conditioning regimen that had at least one mutation detected by NGS [103] utilized MFC and NGS for MRD analysis [103]. The study defined Flow MRD “high” if day +30 level was at least 0.1% and “low” if level was less than 0.1%. “Mutation positive” was defined as persistence of mutations at day +30 and “mutation negative” when mutation(s) were no longer detected. During a median follow up of 15.9 months, relapse occurred in 19.1% of patients. Two-year OS probability was 89% for the flow low and mutation negative subgroup compared to 60% for the flow high and mutation positive subgroup. Probability of two-year—OS in patients that were flow low but mutation positive was 69.2%. The two-year PFS was substantially worse (20% compared to 79%) in those with both flow high and mutation positive compared flow low and mutation negative subgroups.

FIGARO was a phase 2 randomized control study of reduced intensity conditioning regimens that included patients with both MDS and AML. The patients with MDS were required to have International Prognostic Scoring System (IPSS) int-1 with more than 5% blasts or IPSS int-2/high with less than 5% blasts. The MRD assessment was based on MFC with samples from Day 42 up to Month 12 following alloHSCT [155]. Sixty-six patients with MDS had MRD assessment post alloHSCT. Nine of the 66 patients with MDS were found to be MRD-positive in the post-transplant setting. MRD-positivity was associated with inferior overall survival (*p* = 0.0028) and relapse free survival (*p* < 0.0001), although this analysis was also not stratified according to underlying hematologic malignancy (MDS vs. AML). Notably, MRD positivity at day +42 heralded impending relapses, as more than half of these patients relapsed within 8 weeks. Interestingly, full donor T-cell chimerism was associated with lower rates of post-transplant MRD-positivity in this study.

Simultaneous chimerism analysis and post-transplant MRD monitoring via NGS was performed in 14 patients with MDS [48]. Three of the four patients who ultimately relapsed had decreasing donor chimerism and increased mutant allele burden prior to relapse, while one patient was found to have decreasing donor chimerism in the absence of reappearance of initially detected *NRAS* mutation prior to disease relapse. Unfortunately, the mutational analysis of this patient at relapse is not available.

Relapse following alloHSCT has been associated with sub-clonal expansion and mutational evolution in patients with MDS [156]. Exome sequencing identified founding clone mutations as early as 30 days after alloHSCT. Post-transplant MRD detection may inform subsequent management decisions, although the degree of benefit of these therapeutic interventions in patients with MDS remains unclear.

Prospective trials utilizing MRD analysis to guide pre-emptive therapy have also included very few patients with MDS. In the RELAZA trial, 3 patients with MDS were treated with Azacitidine after CD34+ donor chimerism decreased to <80% in the post-transplant setting [157]; one patient had second alloHSCT. In the phase 2 RELAZA2 trial, researchers sought to determine if MRD-guided pre-emptive treatment with Azacitidine could prevent or delay hematologic relapse [158]. Many of the patients had undergone alloHSCT. Twenty-six patients with advanced MDS were included in screening. MRD was assessed for up to 24 months by analysis of donor-chimerism in flow cytometry-sorted CD34-positive cells or by PCR (for *NPM1* mutations or *RUNX1::RUNX1T1*, *CBFB::MYH11*, and *DEK::NUP214* fusion genes). Of the five patients with MDS who were ultimately treated with Azacitidine in the setting of MRD-positivity, two patients experienced a response [158].

Alternatively, Mo et al. [151] utilized post-transplant MRD results to guide selection of subsequent immunotherapies. Of the 21 MRD-positive patients, 6 later received donor lymphocyte infusion, 6 were treated with interferon-a, 1 discontinued immunosuppression, and 1 was treated with chemotherapy following detection of MRD. Conclusions regarding the efficacy of these interventions are again limited by small sample sizes.

**Table 1:** Overview of salient studies in MDS and MRD assessments. Studies with particular interest are denoted with asterisk.

Study	Number of Patients Undergoing alloHSCT	Treatment(s)	Method of MRD Monitoring	Limit of Detection or “Cut-Off” for Positive Result	Setting of MRD Monitoring (Pre- and/or Post-Transplantation)	Conclusions
* Ma et al. [135]	103 patients with MDS-EB with pre-transplantation MRD analysis	Patients received at least one cycle of chemotherapy prior to alloHSCT	Multiparameter flow cytometry	<0.05% to <0.01% throughout duration of study	Pre-transplantation	Worse overall survival (OS) and disease free survival (DFS) in MRD-positive patients; higher cumulative relapse rate in MRD-positive patients.
Sallman et al. [140]	Forty patients with *TP53*-mutated MDS were included in the study	APR-246 + Azacitidine	Next generation sequencing	VAF 5%	Pre-transplantation	*TP53*-mutated patients achieving CR/PR and NGS-negativity prior to alloHSCT had improved OS compared to those undergoing alloHSCT in CR/PR with NGS-positivity.
Dillon et al. [141]	48 patients with MDS with NGS results prior to initiation of conditioning chemotherapy	MAC (myeloablative conditioning) vs. RIC (reduced-intensity conditioning) regimens according to BMT CTN 0901 protocol	DNA sequencing using a custom anchored multiplex polymerase chain reaction–based panel including 10 genes	Minimum allele frequency of 0.001%	Pre-transplantation	Those with detectable pre-transplant mutations had increased rates of relapse and decreased OS. In those with detectable mutations, RIC (compared to MAC) was associated with lower relapse free survival.
Sallman et al. [143]	34 patients with higher-risk MDS	Magrolimab + Azacitidine; 5 patients treated with additional MDS-directed therapy prior to alloHSCT	Multiparameter flow cytometry	0.02%	Pre-transplantation	7 of 34 patients were MRD-negative (median OS not reached) prior to alloHSCT. Median survival was not reached in the MRD-negative cohort.
Bejar et al. [144]	87 patients with MDS	48% had <5% blasts	Deep, massively parallel sequencing examining 40 genes	Not reported	Pre-transplantation	After adjusting for clinical factors associated with poor outcomes, *TP53*, *TET2*, and *DNMT3A* mutations detected prior to alloHSCT were associated with worse survival.
Kharfan-Dabaja et al. [145]	89 patients with MDS	82% received Azacitidine prior to alloHSCT; 92% received MAC regimen	Next generation sequencing examining 26 genes	VAF 10%	Pre-transplantation	*TP53* and *IDH2* mutations were associated with inferior 3-year OS in multivariate analysis.
Hunter et al. [147]	16 patients with *TP53*-mutated MDS proceeded to alloHSCT	All treated with HMA prior to alloHSCT	Next generation sequencing	VAF 5%	Pre-transplantation	7 patients achieved *TP53* mutation clearance prior to alloHSCT, although this did not result in a statistically significant survival advantage compared to 9 patients with *TP53* mutation persistence (*p* = 0.1).
* Festuccia et al. [148]	223 patients with MDS, 66 patients with secondary AML also included	76% had <5% blasts at the time of alloHSCT	Multiparameter flow cytometry and cytogenetics	MFC ranged from 0.1% to 0.001%	Pre-transplantation	Patients with identifiable disease by cytogenetics and treated with RIC regimens had worse survival.
Craddock et al. [149]	80 patients with MDS, 164 patients with AML were also included	Patients randomized to fludarabine-based RIC regimen or FLAMSA-Bu (fludarabine/amsacrine/cytarabine-busulfan)	Multiparameter flow cytometry	Approximately 0.02–0.05%	Pre-transplantation and post-transplantation	Detectable pre-transplantation MRD associated with increased 2-year cumulative incidence of relapse, although results were not stratified by disease (AML vs. MDS).
Yun et al. [153]	37 patients with MDS, CMML, or secondary AML proceeded to alloHSCT	Patients treated with various pre-transplant regimens at a single institution	Next generation sequencing including 37 genes	VAF 5%	Pre-transplantation and post-transplantation	Post-transplantation NGS-negativity associated with significantly improved overall survival.
Bernal et al. [150]	38 patients with MDS; patients with AML also included	Majority received RIC regimen	Multiparameter flow cytometry	0.0001%	Pre-transplantation and day +100 post-transplantation	Day +100 MRD-positivity associated with increased relapse risk and worse overall survival, although results were not stratified by disease (AML vs. MDS),
Mo et al. [151]	78 high-risk or very high-risk patients with MDS	Patients who were MRD-positive after alloHSCT received DLI, interferon-α, chemotherapy, or discontinued immunosuppression	Multiparameter flow cytometry and WT1 PCR	MFC 0.01%, WT1 considered positive if >0.60%	1, 2, 3, 4.5, 6, 9, 12 months post-transplantation and at 6 month intervals thereafter	Two-year cumulative incidence of relapse significantly higher in MRD-positive by either MFC or WT1 PCR.
Duncavage et al. [152]	65 patients with MDS, 21 patients with secondary AML also included	58% received MAC regimen	Enhanced exome sequencing	Considered positive if >0.5%	Day +30 and day +100 post-transplantation	Increased risk of disease progression if detectable mutation at day +30 or +100 even after adjusting for conditioning regimen.
Nakamura et al. [154]	14 patients with MDS, 39 patients with AML also included	Received MAC regimen	Digital droplet PCR (ddPCR) and circulating tumor DNA (ctDNA)	0.04% for ddPCR	1- and 3 months post-transplantation	Results were not stratified by disease (AML vs. MDS), but both ctDNA and ddPCR-positivity at 1- and 3 months post-transplantation were associated with increased risk of relapse and death.
* Tobiasson et al. [37]	177 patients with MDS, 89 additional patients with MDS/MPN or AML with dysplastic features and 20–29% blasts included	Conditioning intensity and previous therapies not reported	ddPCR	Cut-off 0.1%	Bone marrow samples at 1- and 3 months post-transplantation, then every 3 months until month 24 or relapse/death; Peripheral blood samples obtained monthly	Bone marrow MRD-positivity preceded clinical relapse in 42/44 patients by a median of 71 days.
Hou et al. [103]	115 patients with MDS	Received MAC regimen	Multiparameter flow cytometry (MFC) and Next generation sequencing	MFC “high” defined as ≥0.1%	Day +30 post-transplantation	Progression free survival significantly worse for MFC “high” and mutation positive patients.
Loke et al. [155]	66 patients with MDS, 121 patients with AML also included	Received RIC regimen according to FIGARO protocol	Multiparameter flow cytometry	0.05%	Day +42 up to month 12 post-transplantation	MRD-positivity was associated with inferior overall survival and relapse free survival, although this analysis was not stratified according to underlying hematologic malignancy. Full donor T cell chimerism associated with lower rate of MRD-positivity.

## 7. Perspectives

The assessment of MRD in MDS is challenging and in need of validation and standardization of the detection modalities and response categories. The implementation of high-sensitivity techniques including molecular assays in the field of MDS monitoring can offer important insights for the clonal trajectory of the disease especially in the alloHSCT setting. The limitations of the different modalities for assessing MRD in both the pre- and post-transplant settings have been well-described in recent articles [65,159,160].

Multicenter prospective clinical trials with multimodality approach to MRD assessment in MDS including different risk subgroups are needed to validate and determine the most clinically relevant detection method/s and MRD-negative response cutoff/s for various clinical scenarios. The impact of pre-emptive strategies to prevent an impending relapse is an area of great interest and may be impacted by further developments and refinements in MRD testing.
